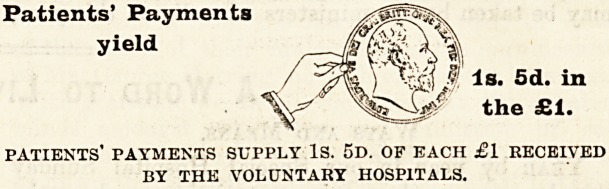# Special Hospital Sunday Supplement

**Published:** 1903-06-13

**Authors:** 


### Special Hospital Sunday Supplement.

### A ROYAL HOSPITAL SUNDAY.

For quite twenty years the active spirits on the Council have looked forward with confidence to the time when it would be possible to raise £100,000 on Hospital Sunday in London. In face of many discouragements and some disappointments they have continuously laboured to secure this result. The Lord Mayor has stated that if the Sunday Fund could raise £100,000, in addition to the means obtained from other sources, the voluntary hospitals would be put in a position of solvency, and all cause for rate assistance would disappear. This statement has given great impetus to the movement, and there are certain special features connected with Hospital Sunday in London in 1903 which afford hopeful signs that the desired goal may this year be reached. The popularity of their Majesties the King and Queen never stood higher than it stands to-day, and they have spontaneously joined hands with the clergy and ministers of religion in the Hospital Sunday movement. This fact alone should secure that at least £100,000 will be forthcoming on June 14th, 1903, but there are other grounds for hope which it is our pleasure to point out.

Although we live in these days under a limited monarchy, the position of the King-Emperor of the British dominions is one of ever-increasing responsibility and labour. To have actively discharged the duties attaching to the position of Prince of Wales for a period of forty years meant an ordeal for the man which few could have passed through with the success and self-restraint which have won for King Edward VII. the respect and warm appreciation of the English people. During these forty years to an ever-increasing extent his Majesty has displayed a keen interest in all kinds of public institutions, and especially in hospitals. The reality and depth of the King's interest in hospitals may be measured by the circumstance, that when Queen Victoria determined to commemorate her Diamond Jubilee by the institution of a great hospital fund for London, his Majesty placed himself at the head of the movement. From the institution of the King's Fund his Majesty took the warmest personal interest in everything connected with it, and he thus rendered direct personal service to the hospitals and made himself more intimately acquainted with the methods of their administration and the character of the work which they do in the metropolis of the Empire.

Again, the visit of King Edward and Queen Alexandra to St. Paul's Cathedral in connection with Hospital Sunday, 1903, is a unique event which should result in immeasurable service to the voluntary system of hospitals throughout the Empire. It is a recognition on the part of their Majesties of their personal interest in the cause of the sick, and may be taken as evidence of the sincerity of their desire to render thanks for the blessings of health. By so doing the King and Queen contribute directly to the aid of the suffering by helping to secure gifts to the hospitals from every one of their subjects in London. For this reason it is a matter for congratulation, that the people are wishful that all ceremonial shall be dispensed with, and that the service in St. Paul's Cathedral may prove an occasion when their Majesties and their subjects will stand before God united in the service of thanksgiving and goodwill to the sick.

Next in importance to the impetus given to the Sunday Fund by the action of their Majesties comes the splendid munificence of Mr. George Herring. By a wise liberality he undertook last year to add 5s. to every pound collected in a place of public worship on behalf of the Hospital Sunday Fund. This year he has gone further, and has offered to give £25,000 providing the clergy and ministers of all denominations put themselves into the work, and move their congregations to contribute £75,000. Last year £62,000 was received on Hospital Sunday by the Hospital Sunday Fund, so that an additional sum of £38,000 will this year be required to bring the whole collections up to £100,000. Mr. George Herring's gift expressed in figures amounts briefly to this: If the clergy and ministers of religion, supported by their congregations, will specially exert themselves, in co-operation with their Majesties the King and Queen, to make Hospital Sunday in London in 1903 memorable for all time, they will raise an extra £24,000 on June 14th, and to that sum Mr. George Herring will add a further £13,425, in addition to the £11,575 he gave last year. To secure this extra £24,000 ought not to be a difficult task for preachers and people. It is a hopeful sign that the Lord Mayor should be able to state that the large banking, insurance, and other limited liability companies have at last come to the decision, that they have the power to contribute out of their profits to the Voluntary Hospitals. No doubt the generosity of Mr. George Herring, and the form in which he has framed his gift, have had much to do with this decision. A knowledge of these facts, too, should stimulate the clergy and ministers of religion, as Mr. George Herring's example has stimulated the business men of the City of London, and if the former will really put their hearts into the effort, the thing will be done. By the adequacy of the sums raised in all the places of worship on June 14th next, London may afford striking evidence of how irresistibly the cause of suffering appeals to men of business, and indeed to all classes of its citizens, when the facts are made clearly apparent and are eloquently presented.

### OVER TWO MILLION SUFFERERS HELPED BY THE HOSPITALS.

### A SINGLE YEAR'S ROLL-CALL OF THE SICK.

How many patients are annually treated in the London Hospitals? The cases which we have sorted out under the various headings comprise those treated at the voluntary hospitals and dispensaries of London—together with the endowed hospitals of St. Bartholomew's, Guy's, and St. Thomas's—and the hospitals of the Metropolitan Asylums Board. Altogether they reach the immense total of *two million and ninety-eight thousand nine hundred and five patients*; and dividing these into men, women, and children, we arrive at the following result:—That of the 2,098,905 patients, 808,212 were men, 711,533 were women, and 578,160 were children.

**Patients Suffering from Surgical Diseases.**—Of the whole number of patients received by the hospitals, *nine hundred and thirty-two thousand one hundred and sixty-six* required surgical treatment. Let us clearly understand what is meant by "surgical diseases." They include not only all accidents such as broken bones, fractured skulls, mangled limbs, and all manner of displacements and crushings of sensitive parts and organs, but also abscesses, ulcerations, cancers, and tumours of all kinds; in short all those injuries which may be produced by accident or pathological process, and which may be dealt with either by hand or instrument. That such a vast number of persons in London suffer from one or more of the injuries thus briefly summarised is not easy to realise. They form a very large army indeed, these 932,166 sufferers.

932,166.
Surgical Patients.

**Patients Suffering from Medical Diseases.**—*Six hundred and fifty-seven thousand six hundred and ninety-eight* persons received medical treatment. By medical diseases is meant those diseases which are situated either as to their source and origin or in their entirety in one or the other of the three great cavities of the body. They include rheumatic fever, pneumonia, pleurisy, bronchitis, diseases of the stomach, bowels, liver, kidney, bladder, and pancreas, every kind of heart disease, many forms of brain injury, dyspepsia, constipation, most nervous diseases, and other ailments, many of them serious and many of them dangerous to life, or at least to the useful existence of the individual. Remember that most of these diseases are out of sight, that the diagnosis of their nature and extent, and the successful treatment of them, is dependent on the doctor's scientific knowledge, and then try and realise that in the hospitals of London over 657,000 persons received treatment at the hands of the foremost physicians of the day, free of cost to the patients themselves. Surely when the hospitals plead for help to us who are in health our answer should be a generous one. Good health is a free gift, and since we have received so lavishly we ought certainly, as a thankoffering, to give liberally.

657,698.
Medical Patients.

**Patients Treated at Special Hospitals for Children.**—*One hundred and sixty-four thousand four hundred and seventy-six* children were sent from homes where they could not be properly attended to for treatment in the hospitals. To see the small face pinched and pain-marked, instead of sunny and dimpled with smiles, the tiny limbs inert and still which should be full of joyous movement and new life, and to hear a low wail of agony from lips which were formed for childish prattle, must pierce the heart of every man and woman worthy the name. Parents who are happy in the knowledge of the noisy nursery at home, whose very lives echo with the pleasant pattering of little feet, cannot withhold their hands. When the children's hospitals appeal for funds to carry on the work of restoring to health and strength the little ones of this great city, the children who will one day take our place as the workers in this land of ours, let the children who are strong and well give to those who are ill and weak.

164,476.
Children.

### THE ROLL-CALL OF THE SICK.—*Continued.*

**Patients Suffering from Eye Affections.**—*One hundred and six thousand four hundred and ninety* persons were treated in the special departments of the general hospitals or by the ophthalmic hospitals of London. The statement that, apart from the blind, nearly a ninth of a million persons have in one year been treated for diseases of the eye in various forms should come home with peculiar force to those who are blessed with sight, and who, too seldom we fear, give one thought to what the loss of it would mean to them. It is certain that very many of these cases must have entailed terrible suffering, and many doubtless would have terminated in total loss of sight but for the skilful treatment they have received at the hospitals. Who can say how many have been saved from becoming practically helpless in the world?

106,490.
Eye.

**Diseases of Women and Motherhood.**—*Seventy-six thousand one hundred and thirteen women* were treated for the special diseases of women at the metropolitan voluntary hospitals, the greater portion attending the hospitals for women and the lying-in institutions. Apart from the diseases to which all are liable, women have to face others peculiar to their sex entailing great suffering, resulting often in permanent or partial disablement. Here it is not only our sympathy which is appealed to, but our patriotism as well. Here there is an actual demand for the payment of a debt we most justly owe. The very heart and strength of the nation lies in the home life, and the soul of the home life is the woman—the mother. The majority of us are to-day what we are because of the influences brought to bear upon us in the home.

76,113
Women.

**Patients Suffering from Diseases of the Ear and Throat.**—At the special hospitals or special departments devoted to these diseases *forty-eight thousand six hundred and seventy-seven* were treated. It is startling to find that so many persons required treatment in the special hospitals for ear, nose and throat. The affections and diseases of these organs, which are intimately connected, involve temporary and often permanent impairment of hearing, swallowing, and breathing. These functions are performed with so little effort on our part that, unless experience has taught us, it is difficult to understand what it would mean to us if we suddenly had to suffer from one or other of these affections.

48,677.
Ear and Throat.

**Patients Suffering from Consumption.**—*Forty-four thousand one hundred and eighty-six* patients suffering from phthisis or consumption were treated at the consumption hospitals of London during the year. The very word consumption makes us afraid. There are few of us who have not seen something of its ravages, of its cruelty. Truly may consumption be called the curse of our climate. It respects neither persons nor estate, neither rich nor poor, the old or the young.

44,186.
Consumption.

**Patients Suffering from Diseases of the Skin.**—During the year *thirty-six thousand and seventy-four* persons were treated for skin diseases in London. It is, perhaps, more difficult to bring home to people the claim which sufferers from skin diseases have upon their sympathy than it is in any of the other classes of disease which we are considering. There is not, as a rule, the pain, nor the danger to life, nor even such risk of permanent disablement as is the case with many of the others; but let us remember what the result would be were there no hospitals for the sufferers to go to.

36,074.
Skin.

**Patients Suffering from Paralysis and Diseases of the Nerves.**—*Seventeen thousand and thirty-five* stricken with paralysis and diseases of the brain received treatment at the hospitals devoted to these maladies. To workers busy with hand and brain these sufferers must particularly appeal. It is impossible to disassociate nervous breakdown from the toil and hurry of existence, especially in a vast centre like London. It is appalling to think that at any moment any one of us may be struck down suddenly, perhaps without the slightest warning. No disease is more sudden than paralysis, surely none more pitiful.

17,034.
Paralysis.

**Patients Suffering from Fever.**—The number of persons who suffered from various forms of fever during the year was 15,990. This figure is however a misleading one, because the term fever includes much besides the class of fevers which are usually removed to these hospitals. Measles, for instance, prevails in London to such an extent that more deaths occur from it than from scarlet fever. The excellent service rendered by the London Fever Hospital entitles it to the gratitude of all householders.

15,990.
Fever.

So this great army of sufferers, numbering over two millions, claim our sympathy and our help year by year. To the strong, to those in health who are able to provide for those dependent upon them, to those who know what ill-health means, who have suffered from disease of one kind or another, and who, either in the hospital or under the skill and care of the doctors and nurses trained in the hospital, have been restored to health and usefulness, we confidently appeal on behalf of the London hospitals.

### HIS MAJESTY THE KING.

THE interest which the King takes in hospitals has been patent to all since the establishment of the Prince of Wales's (now King Edward's) Hospital Fund in the year of Queen Victoria's Diamond Jubilee, but perhaps it has never been clear to the multitude why it was with this form of memorial that the present occupant of Britain's throne chose to associate his name instead of any other of the thousand and one worthy objects which would have welcomed his patronage. To do this it is necessary to look back to the beginning of his career, and see what things attracted him in his early manhood. Doing this, we see that King Edward, while he was still an undergraduate at Cambridge, became patron of Addenbrooke's Hospital there. This is rather notable. It is quite easy to understand how this young gentleman, possessing all the tastes of his fellow students, should have associated himself with the many athletic institutions connected with the University town. But our present King extended his patronage not only to these, but to the local hospitals, institutions the value of which is rarely appreciated by those who have always enjoyed good health. Thus early in his career do we find the name of Edward VII. connected with these palaces of pain with which it is now and will ever be conspicuously associated. In the year of his marriage one of his social duties was the opening of a bazaar in aid of the funds of the Radcliffe Infirmary at Oxford—thus showing his interest in the hospitals in each of the university towns where he had studied—and in that year he became patron of no fewer than seven hospitals. Year by year the list has extended, as hospital work itself has grown, and at the present time there are in London alone 31 hospitals of which he is the patron.

The formal patronage of royalty has often enough been given by earlier monarchs, but King Edward, as his study of our medical charities has extended, has ever become more and more careful to give his name only to institutions which deserve support. He makes inquiries of responsible advisers, and he also investigates for himself. One incident out of many will show that the King personally inquires into the condition of the hospitals to which he gives his patronage. A certain hospital aspired to that honour, but the response to its request was long in coming. One day a private carriage—not a royal one—drove up to the hospital, and out of it stepped a gentleman. It was the King, then the Prince of Wales, come without warning to see the place in its everyday condition. The Royal visitor was conducted not only through the wards but through every part of the building, and inquired as to every detail. Happily, the hospital could stand the test, and not long after the welcome announcement of the Royal patronage was received.

His Majesty has indeed educated himself on the subject of hospitals. He knows what they are, and he knows what they should be. He knows them not merely on the philanthropic side, but on the administrative one also—a side which is largely hidden from the general public. Where he has become president of an institution he has fulfilled the duties of the position, sitting at the court of governors and informing himself regarding the details of management. Thus, in April 1867 his Majesty became president of St. Bartholomew's Hospital. Before doing so he had, in that same month, visited the hospital twice, and after he accepted the position he was present, usually accompanied by the Princess, on almost every festival occasion connected with the institution. He has also been present at each court of governors when business of importance had to be considered, the last occasion being on July 3rd, 1900, when the subject under discussion was the proposed purchase of ground from Christ's Hospital for the enlargement of Bart.'s. When at his accession his Majesty resigned the presidency of the hospital he became its patron. Not every president of a hospital is as faithful in attending to its business as King Edward showed himself during the time when, as Prince of Wales, he was president of St. Bartholomew's.

Perhaps the most conspicuous example of the benefit which an interest taken by the King in an institution will bring in its train is that of Guy's Hospital. For 150 years Guy's was self-supporting, but its revenues, derived from land, had declined to half their former amount, while the demands upon it had largely increased. In 1896 it was resolved to make a special effort to raise a re-endowment fund of half a million. An appeal was issued by Mr. Gladstone, then the senior Governor of the hospital, by which a considerable sum was brought in, and which helped to arouse the interest of the (then) Prince of Wales. It was resolved to hold a festival dinner, and at this dinner his Royal Highness promised to preside. But the honour was to be worked for. A letter from Sir Francis Knollys expressed clearly what the Royal patron expected to be done under the circumstances :—"The Prince of Wales trusts that you will be able to enrol among your stewards prominent representatives of the great commercial and financial interests of the whole nation." In the idea here expressed for the first time, one may fancy that one sees the germ of that Hospital Fund with which the King's name will be for ever connected. At the moment the benefit of it to Guy's was enormous. The hint given was followed up, the most prominent men in the City were aroused to interest in the hospital, and the sum raised in response to this appeal was £167,528, besides several thousands of pounds in annual subscriptions, which if capitalised, would make the capital sum announced at the dinner well over £300,000. Without minimising in the least the value of the previous appeal to the charitable public, one cannot but see that the personal interest of the Prince of Wales, as our King then was, and the advice which he gave, largely brought about this remarkable result—a record among hospital dinners and the money raised by them. Nor did the impetus given by his Majesty to the cause of Guy's end at the moment. How successful and permanent it has been is evidenced by the fact that of the half million wanted only £85,000 now requires to be raised to complete the re-endowment. After this dinner the King became President of Guy's, and retained that position until he came to the throne, when his place was taken by the present Prince of Wales.

Whether or not we are right in tracing to his Majesty's utterance on the occasion of the festival at Guy's the germ of the King's Hospital Fund, it is certain that it was not long afterwards that this fund came into being. Through King Edward's personal efforts the moneyed classes were brought to interest themselves in the hospitals of London, and to contribute to them, not casually and spasmodically, but regularly setting aside a definite sum for this purpose. It has brought men to whom hospitals were only a name to take a genuine interest in their welfare, and to appreciate by personal investigation the work they do. The King had studied the question first, and the King had shown the way.

It may be that even those who support the King's Hospital Fund do not appreciate its national importance. As a charity it appeals to all; there are few so hard-hearted that they would not give something to soothe a sick bed. But beyond that lies the importance to the nation of keeping its workers in health, and restoring them when they have fallen by the way, and the imperial duty of training in our hospitals the doctors and nurses who, wherever the British flag flies, shall maintain and restore the health of King Edward's subjects. This is the work of the hospitals; to help this work that Fund exists with which the King's name is so closely associated, and the care for the sick and suffering of the nation which has marked his career from his youth up, and has grown and strengthened with his years, will ever shine as a bright particular jewel in the crown of Edward VII.

### HER MAJESTY THE QUEEN.

WHEN Princess Alexandra of Denmark came to England to become the bride of the Prince of Wales, her beauty charmed every eye, and her grace won every heart. But though the beauty has been retained in a way that must make other women envious and the grace has never been lost, these outward gifts alone would not have sufficed to explain her unexampled popularity. Beautiful princesses have often enough lost the love of their people and alienated their adherents. But Queen Alexandra's place in the hearts of the people has grown more secure as years have passed, and their love has joyfully set her up as an example to womanhood. In her home, in her Court, in art, in philanthropy, she has set up an ideal for others to follow, in their degree, and illumined every place where she has been set, every duty that has fallen to her lot, not only with the hearth-fire of sympathy and affection, but with the clear sunlight of a high and far-seeing intelligence.

In most accounts of her Majesty, stress is laid upon her gentle and sympathetic disposition. All that could be said on that side of her nature is true, but it does not tell all the truth. Queen Alexandra's gentleness, her sympathy with sorrow and suffering, would not have raised her to the pinnacle on which she stands in the esteem of the nation if it had not been inspired by knowledge and wisdom. The recent incident of the royal lady, going from her palace to visit the new dwellings that were built for her humble fellow-citizens, and observing at once the insufficiency of the cupboards supplied, shows just that practical sagacity which a life like hers, sheltered from the material cares of existence, might be expected to stifle. The same clear-sighted wisdom may be seen in the way she has identified herself with that most successful effort to teach women the virtues of thrift and self-help, the Royal National Pension Fund for Nurses. In that the Queen has shown not merely a kindly interest in hard-working and self-sacrificing women, but a desire to encourage them in a path of wisdom for which women as a rule have very little inclination. Again, in giving her name to one of the first homes for working gentlewomen—the Alexandra House for Art Students—her Majesty has expressed her comprehension of the needs of that feature of our time, the entrance of cultured women into the arena of independent and self-supporting life. Such acts as these show an appreciation of the economic conditions of life among the people of her adoption, and especially of that of women with which, it may be, Queen Alexandra is not always credited.

Her Majesty's philanthropy, though inspired, as all true charity must be, by the heart, is not a mere matter of impulse. If it were, one might make a list of the institutions to which the Queen subscribes and of which she is patroness, and there make an end. But those who come into contact with her in the management of her charities know that the money which her Majesty gives is only a part of her contribution to any good work which commends itself to her. Beyond this is the gift of time and careful inquiry into its conditions, and thoughtful consideration of the best means to help the beneficiaries, not only at the moment, but permanently. One of the problems of our day is how to prevent rural depopulation and the consequent over-crowding of our towns. The Queen, coming from a country which knows how to make country life profitable, perceived the importance of dairy-farming, and has at Sandringham a dairy in which nothing less than perfection is aimed at, and which is an object lesson to others. But the monotony of country life is one of its drawbacks, and those who think they have higher gifts are inclined to leave home to find scope for their talents, often, alas! to meet only with the bitterest disappointment. But for those who live near Queen Alexandra's country home there is the Sandringham technical school, where artistic talent may find an outlet in various forms of decorative work. Naturally a royal example such as this is readily followed, and we owe it to Queen Alexandra in large measure that every part of the United Kingdom has now Home Industries Associations which are both pecuniarily profitable and intellectually helpful to the residents of country districts.

Indeed, it is to a large extent to the example which her Majesty has set that women of all ranks have begun, not only to interest themselves, but to inform themselves on questions affecting the welfare of the people. The Queen is deeply interested in questions of nursing and hospital work, and now in every district we find ladies acting on the committees of nursing associations, and working in one way or another for the local hospital, while in London several hospitals have ladies' associations, presided over by some lady of high and even royal rank, who by their efforts try to win contributions to its funds, and personally help the patients in matters outside the strict limitations of hospital treatment, by visiting the wards, by gifts of needed clothing, and by efforts to provide employment when health is restored. Active, intelligent philanthropy has become fashionable in our day because the Queen is an active and intelligent philanthropist.

But Queen Alexandra, as consort of the ruler of the greatest empire in the world, holds an exceptional position. As in the ultimate resort, all things must be settled by ordeal of battle, the King is, *ipso facto*, head of the Army. That being so, it falls to the Queen to take her place at the head of that section of our defenders whose mission is to save life and restore the sick to health. This task Queen Alexandra has nobly fulfilled. When the African war broke out she came forward to do all in her power to heal and restore our sick and wounded soldiers. Nurses chosen by herself were sent out to the front, and in every detail of their equipment and their work, the Queen took an active and personal interest. Out of her own purse she equipped the hospital ship, *Princess of Wales*, and gave her personal attention to the arrangements for the comfort of its burden of sick and wounded. The same personal interest was shown in the hospital for officers at Sandringham. Womanly tenderness and queenly patriotism united in this work. The accession of Edward VII. gave even larger scope to the task. Queen Alexandra put herself at the head of the Imperial Nursing Service, and took the charge of the sick soldiers and sailors of the Empire as her peculiar care.

In the fullest way, therefore, does Queen Alexandra share the responsibilities of the King's position. In the earlier days of her life in England her charities were very naturally the outcome of her home life. Institutions for the help of children and women who were afflicted by poverty or suffering appealed to her with peculiar force. Nor have these ever been neglected. But, as years went by, the Queen has realised how for deep-seated evils no empirical remedies were of use, and devoted more and more thought to the task of raising those whom she sought to help from their pitiful condition and giving them permanent and practical help. More and more has she learnt that it is impossible to isolate one form of distress and relieve it while leaving others untouched. More and more has she realised as her share of the responsibilities of Empire the duty of aiding all those who look to her for example and guidance, as well as for help, to reach the best they can attain.

Queen Victoria, the mother of her people, shared and sympathised with all their joys and sorrows, but, shadowed by years and griefs, she dwelt apart from them, beloved, revered, but yet remote. But Queen Alexandra has been for nearly forty years in the forefront of social life, observed, studied, imitated by all, an example of purity, of charity, and of beneficent wisdom. Princess of Pity, truly, but also one who will go down to history as the Queen, who by the tenderness of her sympathy, the wisdom of her charities, and the living reality of her example in good works for the least fortunate amongst us, has brought new life and purity everywhere into the field of charity. To her Majesty we owe the fact, that those charities which soothe and heal and bless, no longer, merely lie scattered at men's feet like flowers. They are each and all becoming vigorous plants, instinct with life-giving power to the thoughtful of all classes, who, in ever-increasing numbers, like Queen Alexandra herself, are giving of themselves in personal service to the cause of suffering, and distress, everywhere throughout the Empire.

### QUEEN ALEXANDRA'S SPECIAL CHARITIES.

THE charities of Queen Alexandra are far-reaching and many-sided, but there are certain institutions with which she is peculiarly associated, and which enjoy a special amount of her time and thought. For various reasons she has been drawn towards these, and when once her Majesty is interested in a good work or a beneficent institution she does not lose sight of it. One of the places thus honoured is the Alexandra Hospital for Children with Hip Joint Disease, in Queen Square—one of the most pathetic hospitals in London, where little sufferers pass long months, or even years, in pain, but where patience and care are able often to send them forth, halting, yet useful members of society. The Queen makes a point of her personal association with this institution. "This is *my* hospital," her Majesty said to the present matron, the first time she visited it after that lady's appointment, and explained that it was at her own desire that it bore her name. It is no casual interest that the Queen shows in the hospital and its inmates. When, on Palm Sunday of last year, she visited it, instead of merely walking through the wards as many visitors are content to do, she had beds turned down and the condition of each little sufferer explained to her. Again, after her visit to Denmark this year, she returned to Sandringham, one of her first acts was to send to the Alexandra Hospital a large quantity of primroses

**Metropolitan Hospital.**

**Miller Hospital.**—£20,000 to extend hospital and nursing home.

from the Sandringham woods—country flowers for the little prisoners of pain who were pent in town. Besides visiting the hospital, her Majesty takes a deep interest in the administration and management of it, and likes to be kept informed on points affecting its prosperity.

It is a good many years since a lady belonging to her Majesty's household, who was much interested in the Brompton hospital and had great talent for music, used to come and sing to the patients. On one occasion this lady arranged a concert, but she was very reticent as to who were to assist her. Only a few hours before the entertainment was to begin did she tell the secretary that the (then) Princess of Wales, and the Princesses Louise, Victoria, and Maud, intended to come and use their talents for the pleasure of the invalids. No one but the patients and the officials of the hospital were permitted to be present, but these were made to feel quite at ease by the unaffected simplicity and kindness of the royal ladies' demeanour. On that occasion the Princess took part in a duo for two pianos with her daughters, and also accompanied the Princess Louise, now the Duchess of Fife, when she sang. When the visits were repeated, a whisper of them reached the outside world, and the illustrated papers composed pictures to represent the scene, for the visits were strictly private, and Queen Alexandra was very desirous that they should be so regarded.

When the African war broke out, Queen Alexandra, besides taking a keen interest in all that was done both by the Government and by voluntary effort to minimise the sufferings of the sick and wounded, herself bore the expense of sending out a number of nurses. With these nurses she kept in personal touch. She sent gifts to each, she kept up a correspondence with them, and through them also provided comforts of various kinds for their patients. Her knowledge of the state of things regarding the medical and nursing departments of the army was thus more than a mere official one. To the equipment of the hospital ship, *Princess of Wales*, she gave the same personal attention. Nor did her care end there, for she established a hospital for wounded officers at Babingley, on the Sandringham estate. The house devoted to this purpose had formerly been reserved as a hospital for the estate, but was now let. That the tenant might suffer no inconvenience, their Majesties had a cottage built for him before taking over the old hospital and having it fitted up for its new occupants. The arrangements for the comfort of the patients were all of the most perfect kind, carried out under her Majesty's personal supervision, and many of the pictures and ornaments were put in their places by her own hands. When the royal family were in residence at Sandringham they frequently visited the patients, and the Queen personally conducted several of them over Sandringham House, while all could go, when they were well enough, to see the gardens, stud farm, etc., in which they naturally took a keen interest. As a preference was given to colonial officers as inmates of the hospital, it can easily be understood how the story of the Queen's interest in those who had suffered in the service of the Empire was wafted around the world. An autograph book in which each patient set down a summary of his army career and inserted his photograph, naturally contained also many expressions of appreciation of a kindness that could not but quicken loyalty into devotion.

But perhaps Queen Alexandra's most important contribution to the well-being of her adopted nation was the introduction of the Finsen light treatment for lupus. If her Majesty had done nothing else, this deed should immortalise her name. When on a visit to Denmark, the Princess of Wales, as she was then, heard of this new treatment and visited the Finsen Institute at Copenhagen, where she saw it in operation. So impressed was the Princess with the value of the treatment that she purchased a Finsen apparatus and presented it to the London Hospital. Strange as it may now appear, the hospital was not eager to accept the gift. The cost of maintaining the light is considerable, and the expense was feared. But the royal lady pressed the matter, and the light was installed, with what advantage, not only to the poor who were suffering from a loathsome disease, but to the institution, may be inferred from the fact that not only has the second large light been given by another donor, but endowments amounting to nearly £20,000 have been received for the maintenance of that which is now proudly called "Queen Alexandra's Light Department." Nor did her Majesty's interest end with the presentation of the light. When, last autumn, she heard of an improved Finsen lamp, she immediately bought one for the hospital. So keen is her Majesty's interest in the work that photographs of severe cases are kept for her inspection, in order that she may judge of the progress of the cure.

Such are some of the charities to which Queen Alexandra has given not merely sympathy and help, but active personal service. To tell the whole tale of her good deeds would take far more space than is at our disposal, but this brief account of those to which the Queen has given her thought, her time, her actual work of mind and body, will prove how well deserved is the name which grateful hearts have given her—the Princess of Pity.

**Royal Westminster Ophthalmic Hospital.**

### A Great Opportunity for the Clergy and Ministers.

The Hospital Sunday Fund everywhere in the early days of its establishment and for years afterwards owed its success mainly to the zeal and energy thrown into the work by the clergy and ministers of religion. They realised the privilege of putting themselves into this good work for the sick, and were inspirited by the feeling that here was a common platform on which those of every creed and of none could unitedly stand before God and offer collectively a thank-offering to the Giver of all good for the blessings of health. It was the custom in Birmingham, where the Hospital Sunday Fund was first organised by the late Canon Miller, D.D., for a great number of people and many families to attend a place of worship on Hospital Sunday each year in order to give their offerings to the relief of the sick in the hospitals. Very many people who seldom or never went to church made a point of going there on Hospital Sunday, and the congregations were the most crowded of the year. This, no doubt, inspirited the preachers, and few of them failed to take advantage of the occasion to put their best work into the sermons for that great day.

Habit may become second nature, however, and as the sense of novelty wears off routine may gradually destroy the most sacred enthusiasm. So in a vast city like London preachers and people have, we fear, lost much of the zeal which Hospital Sunday once created in the cause of the hospitals. For years the amount collected in the places of worship remained nearly stationary, and although the number of contributing congregations increased, the total collections showed little, if any, improvement. Last year, however, Mr. George Herring came forward and offered to add five shillings to every sovereign given in a place of worship on Hospital Sunday. The result was most encouraging, for the collections in places of worship increased by £10,000 compared with the average of previous years. Once again Mr. George Herring, with really splendid munificence, repeats his offer, and states that if £75,000 can be raised through the churches and chapels he will give £25,000 in addition to raise the total to £100,000, the sum required from this source to adequately finance the voluntary hospitals of the metropolis. Further than this, their Majesties the King and Queen have associated themselves with the Hospital Sunday Fund, and will attend the service at St. Paul's on Hospital Sunday, 1903, so that the monarch and his people may in the days of health be united in God's house when offering their gifts to the hospitals. Such an occasion is unique in history, and is one which should stir the soul of a people and set a theme which may well move the eloquence of preachers and poets.

In the past many of the clergy and ministers of religion have stated that they were unable to do as much as they otherwise would because they lacked encouragement from the laity. This year, in view of the action of their Majesties and the munificence of Mr. George Herring, no such excuse can be urged. With gratitude we recall the fact that the greater minds amongst the clergy and ministers of religion have always waived aside such excuses as unworthy of their sacred office and unjust to their congregations and their own influence over them. By devoted personal service for weeks before each Hospital Sunday, they have spent themselves in working for the sick. Thus the Rev. Prebendary Ridgway, Canon Fleming, the Chief Rabbi, and many others have continuously increased the sum given by their congregations each year on Hospital Sunday. We hope, therefore, that this year every preacher will take infinite pains to do his best to secure the utmost success in their churches and chapels. It takes a soul to move a body, and we hope the clergy and ministers of religion will give proof of this fact by the whole-hearted energy they will bring to bear on the work of Hospital Sunday. The meetings of the League of Mercy throughout London last winter prove that the heart of the people is sound on this question. With the example of their Majesties before them, and of generous laymen like Mr. George Herring, can it be believed that God's ministers will any of them be content to be indifferent? or to do less than their best for suffering humanity?

There is another side to which we may perhaps briefly refer. In recent times the Nonconformist bodies have been very much in evidence; censuses of Church attendance in the Metropolis have been taken with the apparent object of showing that the Nonconformist places of worship are better attended than those of any other religious body, and efforts have been made to prove through the press that the Church of England was never weaker numerically than it is to-day. In such circumstances the contributions to the hospitals in places of worship becomes more than usually interesting. On page 613 of the "Official Yearbook of the Church of England, 1903," a table is given showing the contributions of the several denominations to the hospitals on Hospital Sunday. From this it appears that in the year 1902 the Church of England contributed £36,822 out of a total of £46,362 raised in that year. The

### LONDON HOSPITALS OF WHICH THE QUEEN IS PATRON AND THEIR NEEDS.

*K.* indicates that the King is Patron as well.

**Alexandra Hospital for Children.**

**Cheyne Hospital.**

**Chelsea Hospital for Women.**—Increased donations and annual subscriptions to keep out of debt and pay off £4,000 mortgage on hospital.

*K.* **City of London Hospital for Chest.**—New nurses' home.

*K.* **East London Hospital for Children.**—An isolation ward. A nursing home. Structural alterations.

**General Lying-in Hospital.**

*K.* **Hospital for Consumption.**—Gifts in money to discharge £2,500 deficiency. Better support from subscribers.

**Hospital for Diseases of Skin.**

Congregationalists contributed £2,136, the Jews £1,396, the Baptists £1,024, the Wesleyans £1,153, the Presbyterians £1,395, the Roman Catholics £573, and the Unitarians £338. Surely when everything has been said that can be said in explanation of these figures there is room left for reflection on the part of every Nonconformist body, and we hope that steps may be taken by the ministers of religion this year, and that a supreme effort may be made to induce the people of every communion to contribute adequately to the hospitals on Hospital Sunday 1903. Rivalry in good works is an admirable thing, and it would be especially welcome and appropriate in the case of Hospital Sunday 1903, when the monarch and his people will unitedly take part in the services throughout London for the succour of the sick.

### A WORD TO LIVING LONDONERS.

### WAYS AND MEANS.

YEAR by year in our Special Hospital Sunday Supplements we have given statistics to show the proportion of the money given for the care of the sick in London by (1) the living, namely, the present inhabitants of the Metropolis, (2) by deceased benefactors and (3) by the patients themselves. We have pointed out how inadequate is the sum contributed by the living to insure the good work which is done by our hospitals being maintained. The latest complete returns are not calculated to make us particularly satisfied with ourselves. We find that in this year (1901), including St. Bartholomew's Hospital, the voluntary contributions amounted to 8s. 2d. in the pound, which, added to the 1s. 5d. received from the patients themselves, bring the amount given by the living up to 9s. 7d. in the pound, a decrease on the amount received in 1900. When, therefore, Londoners are inclined to feel proud of their exceeding liberality to the hospitals they deceive themselves. That is a mean and contemptible spirit which takes credit for virtue it does not possess. If no such reproach is to rest on London this year it is imperative that all classes should give more liberally to the Hospital Sunday Fund and persuade others to follow their example.

### THE INCOME AVAILABLE FOR THE WORK DONE.

In the year 1901 hospital treatment was provided for about two million and seventy thousand persons, exclusive of the patients treated at the hospitals of the Metropolitan Asylums Board, and the total income received by the London voluntary hospitals and dispensaries for this purpose was £1,146,309, which was derived from the following sources :—

| | | |
|---|---|---|
| Charitable or voluntary contributions ... ... ... | £467,437 | or 41 per cent. |
| Income from invested property | 283,073 | „ 25 „ |
| Legacies... ... ... ... ... | 310,975 | „ 27 „ |
| Patients' payments ... ... | 84,824 | „ 7 „ |

So far as the above figures refer to St. Bartholomew's Hospital they have been confined to that portion of the revenue which is applicable to hospital purposes.

### HOW THE MONEY IS PROVIDED.

The mere statement that such and such an amount of money has been raised from one source or another does not, as a rule, greatly impress people. It is rather the difficulties which attend the raising of it, and the consideration of the sources from which it comes, that enable us to judge whether those who are in a position to give are doing their duty. It is therefore of the first importance that everyone should thoroughly understand where the money comes from to pay the cost of the relief given by the hospitals to the inhabitants of London, and to make this as clear as possible we have prepared diagrams, each

THE DEAD HAND GIVES 10S. 5D. OUT OF EVERY £1 RECEIVED BY THE HOSPITALS.

representing a hand and a coin, which have been drawn to scale to show the proportion of every sovereign contributed by the living, *i.e.*, those who receive the benefits, and by deceased benefactors, many of whom have not only left their money to enable the good work to be carried on, but were also during their lives active workers for the hospitals. With a view to clearness the diagrams have been drawn to represent the proportion of every sovereign given in 1901 by (*a*) the dead, (*b*) the living, and

(*c*) the patients themselves. *The black hand* and the coin held by it represent the contributions from those now dead; *the white hand* represents the charitable contributions from the living, *i e.*, ourselves, the living Londoners; and the smallest coin indicates the amount received from patients' payments.

Of every sovereign received 10s. 5d., or more than *one half, is derived from legacies and the interest on gifts* from deceased benefactors, which have been invested in approved securities; 8s. 2d. out of every sovereign has been *given in charity* by the present inhabitant of London, that is, *the living, for the benefit of whose generation the hospitals exist*; and 1s. 5d. of every sovereign has been contributed by those *who have been actually under treament in the hospitals.* Let us study these figures carefully, and understand why they are so unsatisfactory, why they do not inspire us with confidence. It will be seen that a considerable portion of the 10s. 5d. given by the dead hand is derived from the legacies received during the year—to be exact, 5s. 5d. of it—and this

THE LIVING, *i e.*, THE PRESENT INHABITANTS, GIVE 8S. 2D. OF EACH £1 RECEIVED BY THE HOSPITALS.

source of income must always be fluctuating. As compared with 1900 there is a large increase in the amount received from legacies this year, but it is satisfactory to note the average yield is steadily maintained. Still in 1896 there was a sudden drop of £40,000 and in 1900 one of over £70,000. So the sum given by the dead hand may fail us sometimes, and this should be recognised by the living. A fact like this brings us face to face with the certainty that, unless such deficiency is made up by the increased gifts of the living, the great work of our London hospitals must be seriously crippled.

Now let us see how the gifts of the charitable compare this year with last. The actual amount received is larger than in 1900, but is less than in 1899, and represents only 8s. 2d. in the sovereign as against 8s. 7d. in these two years. As we pointed out last year the 8s. 7d. in the £1 for 1900 was only maintained because the total income of the hospitals was less, and this statement is fully borne out by the drop of 5d. in the £1 this year. The number of patients seeking relief from the hospitals steadily increases, and it is a serious position to contemplate that instead of the gifts from the living growing in

PATIENTS' PAYMENTS SUPPLY 1S. 5D. OF EACH £1 RECEIVED BY THE VOLUNTARY HOSPITALS.

proportion to the work done they are decreasing. It seems obvious that the living are shirking their duty. The amount received from the patients themselves is 1s. 5d. in the £1, as against 1s. 7d. in 1900 and 1s. 3d. in 1899, the actual sum received this year being about £7,500 more than last year.

### THE MEANING OF THE DIAGRAMS.

We most earnestly ask all classes in London to spend a few moments in considering the facts disclosed by these figures. While the people of London make use of the hospitals in ever-increasing numbers, the figures prove that they are not ready to make an adequate return for the benefits received, but are content to trust to the dead hand to make up any deficiency. Unless this lamentable lassitude in our charity gives place to more honest endeavour, the ultimate result is not far to seek. In London to-day there are some hospitals unable to take in the patients asking for admission, sometimes because the hospital is not large enough to cope with the district it serves; occasionally because beds are closed for want of funds. Look at this fact from whatever point we will, it is one to make us ashamed. We boast of being citizens of this rich and powerful centre of the greatest Empire the world has known, yet we allow ourselves to remain in imminent danger of the reproach that in spite of our riches, in spite of our power, we grudge the help our suffering and less prosperous brethren need. After all, it is only a very small sacrifice on the part of each individual that is required, and to plead inability is in ninety-nine cases out of a hundred absolute dishonesty. You cannot afford to give a shilling or two to the hospitals, but you never think twice about giving half-a-crown, probably many times a year, for a place in the pit of a theatre. We can all do something. It is our duty; it should be our pleasure. Whether we like it or not, the care of the sick is entrusted to us, and who can be callous enough to neglect such a trust?

### A YEAR'S WORK IN THE HOSPITALS OF LONDON, 1903.

### NEWINGTON AND SOUTH DISTRICT.

Comprising Battersea, Wandsworth, Tooting, Balham, Streatham, Brixton, Lambeth, Newington, Southwark, Bermondsey, Camberwell, Greenwich, Deptford, Lewisham, Blackheath, Woolwich, &c.

| No. of Beds. | No. of Beds Daily Occupied. | HOSPITALS. | In-patients. | Out-patients. | Total Expenditure. | Income. Charitable. | Income. Proprietary. | Income. Patients' Payments. | Total Income. | Legacies not included in previous column. |
|---|---|---|---|---|---|---|---|---|---|---|
| | | | | | £ | £ | £ | £ | £ | £ |
| 652 | 485 | Guy's ... ... ... ... ... | 7,606 | 116,988 | 93,653 | 44,185 | 33,459 | 4,174 | 81,818 | 24,759 |
| 11 | 8 | Phillips Memorial Homœopathic | 145 | 628 | 918 | 547 | 149 | 322 | 1,018 | ... |
| 25 | 22 | Miller ... ... ... ... ... | 294 | 19,469 | 3,569 | 3,635 | 390 | ... | 4,025 | 2,287 |
| 586 | 445 | St. Thomas's ... ... ... | 6,991 | 67,409 | 98,472 | 6,754 | 51,505 | 204 | 58,463 | 9,480 |
| 269 | 234 | Seamen's ... ... ... ... | 2,545 | 21,313 | 22,017 | 10,978 | 3,726 | 1,029 | 15,733 | 3,782 |
| 66 | 19 | Evelina, for Children ... ... | 257 | 5,493 | 9,274 | 1,706 | 3,151 | 29 | 4,886 | 2,213 |
| 57 | 47 | Home for Sick Children ... ... | 265 | 1,536 | 1,901 | 1,082 | 250 | 249 | 1,581 | ... |
| 24 | 20 | General Lying-in... ... ... | 480 | 1,889 | 4,723 | 1,391 | 2,809 | ... | 4,200 | ... |
| 30 | 17 | Clapham Maternity & Dispensary | 365 | 5,239 | 2,345 | 425 | 1,123 | 881 | 2,429 | ... |
| 42 | 28 | Royal Eye ... ... ... ... | 607 | 19,141 | 3,321 | 2,014 | 194 | 658 | 2,866 | 449 |
| 11 | 9 | Eltham Cottage ... ... ... | 147 | ... | 759 | 754 | 38 | 155 | 947 | 463 |
| 30 | 14 | Beckenham Cottage ... ... | 194 | ... | 954 | 604 | 21 | 114 | 739 | — |
| 18 | 12 | Blackheath Cottage ... ... | 154 | 990 | 1,129 | 845 | 39 | 105 | 989 | 100 |
| 32 | 20 | Bromley Cottage ... ... ... | 361 | ... | 1,580 | 1,544 | 251 | 138 | 1,933 | ... |
| 21 | 16 | Chislehurst, &c., Cottage ... | 233 | ... | 856 | 583 | 7 | 311 | 901 | ... |
| 14 | 6 | Sidcup Cottage ... ... ... | 122 | ... | 577 | 403 | 23 | 192 | 618 | ... |
| 7 | 6 | Shortlands... ... ... ... | 110 | ... | 246 | 234 | 3 | 5 | 242 | ... |
| 18 | 11 | Livingstone Cottage ... ... | 152 | ... | 1,114 | 1,034 | 18 | 34 | 1,086 | ... |
| 10 | 9 | Woolwich and Plumstead Cottage | 81 | 16 | 531 | 385 | 11 | 121 | 517 | ... |
| 30 | 30 | Bolingbroke Hospital ... ... | 378 | 6,042 | 4,475 | 3,723 | 77 | 590 | 4,399 | 1,025 |
| 41 | 23 | St. John's, Lewisham ... ... | 285 | ... | 2,438 | 861 | 131 | 463 | 1,455 | ... |
| 1,994 | 1,481 | | 21,772 | 266,153 | 234,852 | 83,696 | 97,375 | 9,774 | 190,845 | 44,558 |
| | | DISPENSARIES. | | | | | | | | |
| ... | ... | Battersea Provident ... ... | ... | 36,564 | 3,525 | 119 | 67 | 3,388 | 3,574 | ... |
| ... | ... | Blackfriars, Provident ... ... | ... | 1,338 | 274 | 98 | ... | 176 | 274 | ... |
| ... | ... | Brixton, &c. ... ... ... | ... | 4,371 | 730 | 592 | 34 | 84 | 710 | ... |
| ... | ... | Camberwell Provident ... ... | ... | 7,039 | 1,802 | 333 | 155 | 1,412 | 1,900 | ... |
| ... | ... | Clapham ... ... ... ... | ... | 1,435 | 356 | 226 | 26 | 117 | 369 | ... |
| ... | ... | Deptford Medical Mission ... | ... | 2,461 | 320 | 213 | 31 | 77 | 321 | ... |
| ... | ... | East Dulwich Provident ... | ... | 4,977 | 914 | 111 | 6 | 876 | 993 | ... |
| ... | ... | Forest Hill Provident ... ... | ... | 2,237 | 738 | 245 | 3 | 527 | 775 | ... |
| ... | ... | Greenwich Provident ... ... | ... | 2,850 | 651 | 65 | 18 | 597 | 680 | ... |
| ... | ... | Royal South London ... ... | ... | 3,835 | 754 | 562 | 84 | ... | 646 | ... |
| ... | ... | South Lambeth, &c. ... ... | ... | 2,011 | 500 | 361 | 6 | 149 | 516 | ... |
| ... | ... | Walworth Provident ... ... | ... | 730 | 272 | 44 | 17 | 160 | 221 | ... |
| ... | ... | Wandsworth Common ... ... | ... | 1,084 | 248 | 51 | ... | 197 | 248 | ... |
| ... | ... | Woolwich, &c., Provident ... | ... | 2,346 | 425 | 41 | 1 | 327 | 369 | ... |
| 1,994 | 1,481 | | 21,772 | 339,431 | 246,361 | 86,757 | 97,823 | 17,861 | 202,441 | 44,558 |

### WESTMINSTER DISTRICT.—Comprising Westminster City and Liberties.

| No. of Beds. | No. of Beds Daily Occupied. | HOSPITALS. | In-patients. | Out-patients. | Total Expenditure. | Income. Charitable. | Income. Proprietary. | Income. Patients' Payments. | Total Income. | Legacies not included in previous column. |
|---|---|---|---|---|---|---|---|---|---|---|
| | | | | | £ | £ | £ | £ | £ | £ |
| 150 | 132 | Charing Cross ... ... ... | 1,986 | 18,073 | 49,361 | 17,078 | 2,510 | 41 | 19,629 | 7,717 |
| 224 | 180 | King's College ... ... ... | 2,613 | 19,767 | 21,571 | 11,134 | 4,142 | 43 | 15,319 | 6,930 |
| 213 | 158 | Westminster ... ... ... | 2,384 | 22,270 | 20,972 | 15,288 | 3,167 | 11 | 18,466 | 6,954 |
| 154 | 145 | Ventnor, for Consumption ... | 760 | ... | 14,749 | 8,314 | 2,420 | 4,099 | 14,833 | 1,600 |
| 23 | 17 | Grosvenor, for Women & Children | 203 | 4,370 | 2,328 | 1,422 | 94 | 560 | 2,076 | 632 |
| 64 | 39 | Hospital for Women ... ... | 662 | 4,354 | 5,951 | 4,188 | 356 | 1,079 | 5,623 | 1,721 |
| 25 | 20 | National, for Diseases of Heart... | 154 | 2,747 | 2,605 | 1,238 | 96 | 688 | 2,022 | 1,300 |
| 40 | 28 | Royal Westminster Ophthalmic... | 588 | 9,602 | 2,598 | 2,655 | 643 | ... | 3,298 | 116 |
| 50 | 35 | Royal Orthopædic ... ... | 227 | 983 | 2,495 | 1,177 | 400 | 238 | 1,815 | 100 |
| 9 | 7 | Royal Ear ... ... ... ... | 231 | 1,950 | 828 | 300 | ... | 499 | 799 | ... |
| ... | ... | Dental ... ... ... ... ... | ... | 46,805 | 3,094 | 3,144 | 128 | 240 | 3,512 | 308 |
| 27 | 18 | Gordon, for Fistula ... ... | 270 | 924 | 1,769 | 325 | 6 | 1,387 | 1,718 | 225 |
| 27 | 21 | St. Peter's, for Stone ... ... | 481 | 4,346 | 4,528 | 1,496 | 342 | 2,201 | 4,039 | ... |
| 50 | 34 | St. John's, for Skin ... ... | 259 | 7,959 | 3,921 | 1,405 | 3 | 2,572 | 3,980 | ... |
| 53 | 28 | Hospital for Diseases of Throat... | 773 | 10,852 | 4,869 | 1,569 | 59 | 3,687 | 5,315 | 100 |
| 1,089 | 862 | | 11,591 | 155,002 | 141,639 | 70,733 | 14,366 | 17,345 | 102,444 | 27,703 |
| | | DISPENSARIES. | | | | | | | | |
| ... | ... | Public ... ... ... ... ... | ... | 2,153 | 656 | 411 | 229 | ... | 640 | ... |
| ... | ... | St. George and St. James ... | ... | 1,894 | 629 | 461 | 5 | 35 | 501 | ... |
| ... | ... | St. George's, Hanover Square ... | ... | 754 | 543 | 320 | ... | 170 | 490 | ... |
| ... | ... | Western ... ... ... ... | ... | 9,737 | 1,513 | 531 | 429 | 736 | 1,696 | ... |
| ... | ... | Westminster General ... ... | ... | 5,816 | 847 | 507 | 213 | 105 | 825 | ... |
| 1,089 | 862 | | 11,591 | 175,356 | 145,827 | 72,963 | 15,242 | 18,391 | 106,596 | 27,703 |

### ST. MARYLEBONE AND WEST CENTRAL DISTRICT.

Comprising St. Marylebone, St. John's Wood, Bloomsbury, Holborn, &c.

| No. of Beds. | No. of Beds Daily Occupied. | HOSPITALS. | In-patients. | Out-patients. | Total Expenditure. | Income. Charitable. | Income. Proprietary. | Income. Patients' Payments. | Total Income. | Legacies not included in previous column |
|---|---|---|---|---|---|---|---|---|---|---|
| | | | | | £ | £ | £ | £ | £ | £ |
| 70 | 54 | French ... ... ... ... | 875 | 5,009 | 4,792 | 4,173 | 12 | ... | 4,185 | 1,202 |
| 50 | 34 | Italian ... ... ... ... | 600 | 10,111 | 2,471 | 3,157 | 261 | ... | 3,418 | 152 |
| 103 | 73 | London Homœopathic ... ... | 1,031 | 20,749 | 11,833 | 3,232 | 4,028 | 875 | 8,135 | 1,574 |
| 110 | 59 | SS. John and Elizabeth ... ... | 202 | ... | 3,918 | 2,270 | 350 | 1,008 | 3,708 | 887 |
| 341 | 303 | The Middlesex ... ... ... | 4,019 | 41,585 | 53,210 | 22,809 | 9,273 | ... | 32,082 | 30,179 |
| 76 | 68 | Alexandra for Children ... ... | 136 | 320 | 4,199 | 4,075 | 273 | 325 | 4,673 | ... |
| 30 | 29 | Hospital for Incurable Children | 33 | ... | 1,492 | 428 | 132 | 461 | 1,021 | 1,000 |
| 252 | 181 | Hospital for Sick Children ... | 2,236 | 25,310 | 20,022 | 26,416 | 4,018 | ... | 30,434 | 1,792 |
| 20 | 16 | British Lying-in ... ... ... | 414 | 675 | 2,534 | 533 | 1,857 | 64 | 2,454 | ... |
| 74 | 54 | Queen Charlotte's Lying-In ... | 1,394 | 1,204 | 5,602 | 4,723 | 292 | 122 | 5,137 | 1,808 |
| 52 | 46 | New Hospital for Women ... | 619 | 15,351 | 5,996 | 3,433 | 471 | 1,568 | 5,472 | 5,341 |
| 47 | 44 | Samaritan Free ... ... ... | 495 | 6,737 | 5,652 | 4,155 | 270 | ... | 4,425 | 592 |
| 196 | 171 | National for the Paralysed, &c.... | 1,004 | 6,366 | 23,122 | 7,246 | 1,926 | 2,652 | 11,824 | 6,568 |
| Closed | | Hospital for Epilepsy, &c. ... | ... | 613 | 884 | 673 | 28 | 200 | 901 | 2,593 |
| 50 | 18 | West End, for Epilepsy, &c. ... | 177 | 4,180 | 4,254 | 3,848 | 75 | 553 | 4,476 | ... |
| 28 | 17 | Central London Ophthalmic ... | 357 | 12,270 | 1,814 | 1,740 | 40 | ... | 1,780 | ... |
| 13 | 7 | Western Ophthalmic ... ... | 195 | 12,351 | 1,030 | 498 | 173 | 6 | 677 | ... |
| 60 | 59 | National Orthopædic ... ... | 234 | 1,430 | 3,047 | 1,831 | 55 | 843 | 2,729 | 100 |
| 20 | 15 | Establishment for Gentlewomen. | 164 | ... | 2,656 | 1,788 | 143 | 1,035 | 2,966 | 1,050 |
| ... | ... | National Dental ... ... ... | ... | 21,167 | 1,893 | 236 | ... | 1,028 | 1,264 | ... |
| 16 | 10 | London Throat ... ... ... | 658 | 4,557 | 1,398 | 300 | ... | 1,034 | 1,334 | ... |
| 17 | 8 | Metropolitan Ear, Nose & Throat | 275 | 3,123 | 870 | 426 | ... | 369 | 795 | 100 |
| 32 | 23 | Oxygen Hospital ... ... ... | 96 | ... | 1,214 | 513 | 18 | 907 | 1,438 | ... |
| 1,657 | 1,289 | | 15,214 | 193,108 | 163,903 | 98,503 | 23,695 | 13,130 | 135,328 | 54,938 |
| | | DISPENSARIES. | | | | | | | | |
| ... | ... | Bloomsbury Provident ... ... | ... | 711 | 270 | 33 | ... | 205 | 238 | ... |
| ... | ... | London Medical Mission ... | ... | 7,579 | 1,286 | 830 | 75 | 265 | 1,170 | ... |
| ... | ... | Margaret Street, for Consumption | ... | 681 | 471 | 217 | 181 | ... | 398 | ... |
| ... | ... | Portland Town ... ... ... | ... | 1,280 | 163 | 155 | 5 | 10 | 170 | ... |
| ... | ... | St. John's Wood Provident ... | ... | 5,473 | 735 | 451 | ... | 389 | 840 | ... |
| ... | ... | St. Marylebone General ... ... | ... | 4,046 | 882 | 457 | 163 | 203 | 823 | ... |
| ... | ... | Western General ... ... ... | ... | 14,778 | 1,359 | 946 | 48 | 55 | 1,049 | 409 |
| 1,657 | 1,289 | | 15,214 | 227,656 | 169,069 | 101,592 | 24,167 | 14,257 | 140,016 | 55,347 |

### STRATFORD AND EAST-END DISTRICT.

Comprising Bethnal Green, Tower Hamlets, West Ham, Whitechapel, Hackney, Stepney, Limehouse, Poplar, and the East.

| No. of Beds. | No. of Beds Daily Occupied. | HOSPITALS. | In-patients. | Out-patients. | Total Expenditure. | Income. Charitable. | Income. Proprietary. | Income. Patients' Payments. | Total Income. | Legacies not included in previous column. |
|---|---|---|---|---|---|---|---|---|---|---|
| | | | | | £ | £ | £ | £ | £ | £ |
| 130 | 118 | German ... ... ... ... | 1,691 | 25,468 | 10,712 | 6,005 | 2,689 | 475 | 9,169 | 340 |
| 790 | 682 | London ... ... ... ... | 12,524 | 162,147 | 180,790 | 48,994 | 27,665 | 1,580 | 78,239 | 23,641 |
| 50 | 36 | Mildmay Mission Hospital ... | 655 | 11,324 | 3,742 | 2,704 | 589 | 92 | 3,385 | 1,500 |
| 103 | 68 | Poplar ... ... ... ... | 1,294 | 25,217 | 12,489 | 8,241 | 1,133 | 168 | 9,542 | 2,628 |
| 60 | 47 | West Ham, &c. ... ... ... | 790 | 21,404 | 5,945 | 6,606 | 347 | ... | 6,953 | 100 |
| 29 | 16 | Walthamstow, &c. ... ... | 307 | 1,648 | 5,757 | 1,693 | 242 | ... | 1,935 | ... |
| 164 | 124 | City of London for Dis. of the Chest | 927 | 9,837 | 11,780 | 8,909 | 318 | ... | 9,227 | 937 |
| 135 | 118 | East London for Children ... | 2,146 | 32,981 | 9,909 | 7,556 | 1,164 | ... | 8,720 | 3,550 |
| 35 | 23 | St. Mary's, Plaistow ... ... | 453 | 5,905 | 3,267 | 2,924 | 129 | 139 | 3,192 | ... |
| 16 | 12 | East End Mothers' Home ... | 313 | 367 | 2,052 | 1,057 | 629 | 49 | 1,735 | ... |
| 13 | 9 | Canning Town Cottage ... ... | 130 | 1,453 | 941 | 515 | 263 | 187 | 965 | ... |
| 16 | 5 | Passmore Edwards Cottage, T'lb'ry | 100 | 453 | 852 | 1,093 | 29 | ... | 1,122 | ... |
| 1,541 | 1,258 | | 21,330 | 308,204 | 248,136 | 96,297 | 35,197 | 2,690 | 134,184 | 32,696 |
| | | DISPENSARIES. | | | | | | | | |
| ... | ... | Eastern ... ... ... ... | ... | 7,544 | 856 | 181 | 323 | 283 | 787 | ... |
| ... | ... | Hackney Provident ... ... | ... | 1,672 | 277 | 95 | ... | 206 | 301 | ... |
| ... | ... | London ... ... ... ... | ... | 2,106 | 441 | 200 | 276 | ... | 476 | ... |
| ... | ... | Queen Adelaide's ... ... ... | ... | 5,935 | 606 | 444 | 222 | ... | 666 | 50 |
| ... | ... | Tower Hamlets ... ... ... | ... | 3,431 | 620 | 312 | 26 | 150 | 488 | 100 |
| ... | ... | Whitechapel Provident ... ... | ... | 4,495 | 814 | 92 | ... | 718 | 810 | ... |
| 1,541 | 1,258 | | 21,330 | 333,387 | 251,750 | 97,621 | 36,044 | 4,047 | 137,712 | 32,846 |

### ISLINGTON AND NORTH-WEST DISTRICT.

Comprising Islington, Holloway, Highbury, Hampstead, Highgate, St. Pancras, Stoke Newington, Tottenham, &c.

| No. of Beds. | No. of Beds Daily Occupied. | HOSPITALS. | In-patients. | Out-patients. | Total Expenditure. | Income. | | | Total Income. | Legacies not included in previous column. |
|---|---|---|---|---|---|---|---|---|---|---|
| | | | | | | Charitable. | Proprietary. | Patients' Payments. | | |
| | | | | | £ | £ | £ | £ | £ | £ |
| 159 | 138 | Great Northern Central ... ... | 1,941 | 24,536 | 14,540 | 9,921 | 1,326 | 597 | 11,844 | 10,946 |
| 32 | 24 | Hampstead Hospital ... ... | 373 | 2,511 | 3,110 | 3,037 | 8 | 299 | 3,344 | 285 |
| 120 | 85 | London Temperance ... ... | 1,471 | 24,135 | 11,307 | 5,030 | 1,841 | 272 | 7,143 | 3,052 |
| 53 | 43 | North-West London ... ... | 601 | 17,564 | 5,210 | 2,676 | 111 | 46 | 2,833 | 1,150 |
| 73 | 64 | Tottenham ... ... ... | 820 | 17,153 | 5,137 | 6,350 | 111 | ... | 6,461 | 325 |
| 188 | 168 | University College ... ... | 2,693 | 46,856 | 23,567 | 10,937 | 3,770 | 33 | 14,740 | 10,710 |
| 100 | 92 | Mount Vernon for Consumption | 547 | 4,962 | 10,572 | 7,667 | 67 | ... | 7,734 | 700 |
| 14 | 12 | Children's Home Hospital, Barnet | 59 | ... | 545 | 449 | ... | 80 | 529 | ... |
| 200 | 63 | London Fever ... ... ... | 599 | ... | 12,240 | 10,320 | 2,000 | 1,600 | 13,920 | 4,616 |
| 28 | 15 | Invalid Asylum ... ... ... | 200 | ... | 810 | 385 | 126 | 155 | 666 | ... |
| 14 | 11 | Enfield Cottage ... ... ... | 166 | ... | 660 | 484 | 30 | 23 | 537 | 20 |
| 30 | 14 | Memorial Cottage, Mildmay ... | 186 | ... | 1,578 | 330 | 1,003 | 279 | 1,612 | 1,500 |
| 27 | 17 | St. Saviour's Home ... ... | 135 | ... | 2,184 | 1,102 | 8 | 873 | 1,983 | 30 |
| 42 | 36 | Friedenheim Hospital ... ... | 119 | ... | 3,543 | 2,702 | 138 | 265 | 3,105 | 1,375 |
| 28 | 23 | St. Monica's, Brondesbury ... | 57 | ... | 1,676 | 1,067 | 215 | 331 | 1,613 | 500 |
| 25 | 20 | Willesden Cottage ... ... | 233 | ... | 1,447 | 1,434 | 68 | 71 | 1,573 | ... |
| 20 | 8 | Bushey Heath Cottage ... ... | 119 | ... | 803 | 1,372 | 56 | 181 | 1,609 | ... |
| 20 | 19 | Santa Claus Home ... ... | 48 | ... | 672 | 677 | 13 | 123 | 813 | ... |
| 1,173 | 852 | | 10,367 | 137,717 | 99,601 | 65,940 | 10,891 | 5,228 | 82,059 | 35,209 |
| | | DISPENSARIES. | | | | | | | | |
| ... | ... | Camden Provident ... ... | ... | 1,099 | 283 | 30 | ... | 248 | 278 | ... |
| ... | ... | Childs' Hill, Provident ... ... | ... | 1,914 | 353 | 40 | 12 | 300 | 352 | ... |
| ... | ... | Hampstead Provident ... ... | ... | 11,463 | 1,070 | 283 | 29 | 767 | 1,079 | ... |
| ... | ... | Holloway and North Islington ... | ... | 3,280 | 724 | 249 | 41 | 268 | 558 | 54 |
| ... | ... | Islington ... ... ... ... | ... | 11,780 | 1,051 | 280 | 17 | 566 | 863 | 50 |
| ... | ... | St. Pancras and Northern ... | ... | 1,676 | 553 | 228 | 129 | 110 | 467 | 200 |
| ... | ... | Stamford Hill, &c. ... ... | ... | 9,388 | 797 | 565 | 164 | ... | 729 | 111 |
| 1,173 | 852 | | 10,367 | 178,317 | 104,432 | 67,615 | 11,283 | 7,487 | 86,385 | 35,624 |

### KENSINGTON AND WEST DISTRICT.

Comprising Kensington, Paddington, Bayswater, Kilburn, Chelsea, Brompton, Fulham, Hammersmith, Chiswick, Brentford, Acton, Ealing, &c.

| No. of Beds. | No. of Beds Daily Occupied. | HOSPITALS. | In-patients. | Out-patients. | Total Expenditure. | Charitable. | Proprietary. | Patients' Payments. | Total Income. | Legacies not included in previous column. |
|---|---|---|---|---|---|---|---|---|---|---|
| | | | | | £ | £ | £ | £ | £ | £ |
| 18 | 13 | Queen's Jubilee ... ... ... | 200 | 7,939 | 1,568 | 1,540 | 31 | ... | 1,571 | ... |
| 351 | 251 | St. George's ... ... ... | 3,761 | 31,833 | 45,051 | 28,489 | 14,734 | ... | 43,223 | 10,925 |
| 281 | 248 | St. Mary's ... ... ... ... | 3,927 | 45,171 | 29,761 | 12,153 | 2,696 | ... | 14,849 | 12,268 |
| 146 | 131 | West London ... ... ... | 2,141 | 36,573 | 10,931 | 7,533 | 561 | ... | 8,094 | 5,065 |
| 315 | 257 | Hospital for Consumption ... | 1,282 | 7,304 | 32,732 | 12,424 | 6,650 | ... | 19,074 | 26,262 |
| 26 | 14 | Belgrave, for Children ... ... | 205 | 3,969 | 1,116 | 953 | 34 | ... | 987 | 100 |
| 50 | 50 | Cheyne, for Sick & Incurable Chldn | 64 | ... | 2,644 | 1,740 | 471 | 334 | 2,545 | 890 |
| 46 | 32 | Paddington Green, for Children | 589 | 16,049 | 4,863 | 3,682 | 272 | 326 | 4,280 | 1,050 |
| 92 | 76 | Victoria, for Children ... ... | 1,068 | 17,395 | 6,917 | 5,058 | 732 | 410 | 6,200 | 5,300 |
| 52 | 48 | Chelsea, for Women ... ... | 793 | 2,778 | 5,734 | 3,251 | 114 | 872 | 4,237 | 2,685 |
| 105 | 86 | Cancer ... ... ... ... | 732 | 1,709 | 13,015 | 5,114 | 4,002 | ... | 9,116 | 11,643 |
| 135 | 90 | Female Lock ... ... ... | 603 | ... | 5,038 | 2,728 | 28 | 1,924 | 4,680 | 1,134 |
| 16 | 9 | Epsom and Ewell Cottage ... | 97 | ... | 1,103 | 944 | 19 | 217 | 1,180 | ... |
| 30 | 19 | Reigate and Redhill Cottage ... | 275 | ... | 1,251 | 978 | 8 | 217 | 1,203 | ... |
| 16 | 9 | Wimbledon Cottage ... ... | 141 | ... | 604 | 620 | 13 | 134 | 767 | 100 |
| 15 | 8 | Hounslow Cottage ... ... | 153 | 658 | 644 | 440 | 210 | 54 | 704 | ... |
| ... | ... | Acton Cottage ... ... ... | 134 | 575 | 1,080 | 1,054 | 83 | 6 | 1,143 | ... |
| 1,694 | 1,341 | | 16,165 | 171,953 | 164,052 | 88,701 | 30,658 | 4,494 | 123,853 | 77,422 |
| | | DISPENSARIES. | | | | | | | | |
| ... | ... | Brompton Provident ... ... | ... | 1,651 | 363 | 107 | 56 | 200 | 363 | ... |
| ... | ... | Chelsea, &c. ... ... ... | ... | 4,094 | 661 | 365 | 178 | ... | 543 | ... |
| ... | ... | Chelsea Provident ... ... | ... | 1,969 | 254 | 32 | 17 | 165 | 214 | ... |
| ... | ... | Kensal Town Provident ... ... | ... | 880 | 259 | 37 | ... | 243 | 280 | ... |
| ... | ... | Kensington ... ... ... | ... | 3,381 | 1,149 | 1,001 | 81 | ... | 1,082 | 120 |
| ... | ... | Kilburn, Maida Vale ... ... | ... | 2,358 | 452 | 407 | 47 | ... | 454 | 150 |
| ... | ... | Kilburn Provident ... ... | ... | 7,068 | 1,252 | 64 | 12 | 1,200 | 1,276 | ... |
| ... | ... | Notting Hill Provident ... ... | ... | 637 | 250 | 73 | ... | 143 | 216 | ... |
| ... | ... | Paddington Provident ... ... | ... | 2,638 | 533 | 166 | 24 | 326 | 516 | ... |
| ... | ... | Royal Pimlico Provident... ... | ... | 8,150 | 853 | 323 | 16 | 500 | 839 | 100 |
| ... | ... | Westbourne Provident ... ... | ... | 1,384 | 433 | 48 | 45 | 331 | 424 | ... |
| 1,694 | 1,341 | | 16,165 | 206,163 | 170,511 | 91,324 | 31,134 | 7,602 | 130,060 | 77,792 |

### CITY AND EAST CENTRAL DISTRICT.

Comprising the City, St. Luke's, Shoreditch, Finsbury, and Clerkenwell.

| No. of Beds. | No. of Beds Daily Occupied. | HOSPITALS. | In-patients. | Out-patients. | Total Expenditure. | Income. Charitable. | Income. Proprietary. | Income. Patients' Payments. | Total Income. | Legacies not included in previous column. |
|---|---|---|---|---|---|---|---|---|---|---|
| | | | | | £ | £ | £ | £ | £ | £ |
| 111 | 92 | Metropolitan ... ... ... | 1,285 | 40,592 | 14,476 | 10,796 | 758 | 554 | 12,108 | 800 |
| 170 | 144 | Royal Free ... ... ... | 2,157 | 38,013 | 13,491 | 5,636 | 1,247 | ... | 6,883 | 12,039 |
| 80 | 57 | Royal, for Diseases of the Chest. | 779 | 6,906 | 7,308 | 6,571 | 163 | ... | 6,734 | 522 |
| 57 | 47 | North-Eastern, for Children ... | 818 | 19,318 | 6,538 | 6,944 | 244 | 716 | 7,904 | 21 |
| 42 | 23 | City of London Lying-In ... | 524 | 2,242 | 3,700 | 463 | 4,163 | 5 | 4,631 | ... |
| 37 | 34 | St. Mark's, for Fistula ... ... | 385 | 1,185 | 4,677 | 3,062 | 742 | ... | 3,804 | 590 |
| 138 | 74 | Royal London Ophthalmic ... | 1,979 | 32,442 | 11,695 | 9,596 | 349 | ... | 9,945 | 1,454 |
| 45 | 40 | City Orthopædic ... ... ... | 290 | 2,054 | 2,421 | 2,326 | 25 | ... | 2,351 | ... |
| 16 | 11 | Central London Throat and Ear. | 339 | 9,037 | 2,268 | 478 | 76 | 1,608 | 2,162 | 1,168 |
| 696 | 522 | | 8,556 | 151,789 | 66,574 | 45,872 | 7,767 | 2,883 | 56,522 | 16,594 |
| | | DISPENSARIES. | | | | | | | | |
| ... | ... | Billingsgate Medical Mission ... | ... | 2,203 | 350 | 330 | ... | 58 | 388 | ... |
| ... | ... | City ... ... ... ... ... | ... | 6,321 | 1,031 | 1,148 | 86 | ... | 1,234 | ... |
| ... | ... | City of London and East London | ... | 27,325 | 1,758 | 97 | 115 | 2,232 | 2,444 | 90 |
| ... | ... | Farringdon General ... ... | ... | 2,360 | 779 | 735 | ... | 217 | 952 | ... |
| ... | ... | Finsbury ... ... ... ... ... | ... | 12,213 | 1,078 | 714 | 168 | 266 | 1,148 | ... |
| ... | ... | Metropolitan ... ... ... | ... | 5,641 | 865 | 518 | 122 | 250 | 890 | ... |
| ... | ... | Royal General ... ... ... | ... | 2,838 | 748 | 296 | 407 | 78 | 781 | ... |
| 696 | 522 | | 8,556 | 210,690 | 73,183 | 49,710 | 8,665 | 5,984 | 64,359 | 16,684 |

### THE METROPOLITAN HOSPITALS.—A SUMMARY OF THE WORK DONE IN 1902.

It will be seen from the following summary that the Voluntary Hospitals and Medical Charities of London, during the twelve months ending December 31st, 1902, relieved over One million seven hundred and seventy-five thousand patients at a cost of £1,161,133. The Ordinary Income only amounted to £867,569, leaving a deficiency of £293,564 on the year's work. The legacies received in 1902 amounted to £290,554, being £24,776 more than the amount received in 1901.

| No. of Beds. | No. of Beds Daily Occupied. | HOSPITALS AND DISPENSARIES. | In-patients. | Out-patients. | Total Expenditure. | Income. Charitable. | Income. Proprietary. | Income. Patients' Payments. | Total Income. | Legacies not included in previous column. |
|---|---|---|---|---|---|---|---|---|---|---|
| | | | | | £ | £ | £ | £ | £ | £ |
| 1,994 | 1,481 | Newington and South District... | 21,772 | 339,431 | 246,361 | 86,757 | 97,823 | 17,861 | 202,441 | 44,558 |
| 696 | 522 | City and East Central District... | 8,556 | 210,690 | 73,183 | 49,710 | 8,665 | 5,984 | 64,359 | 16,684 |
| 1,089 | 862 | Westminster District ... ... | 11,591 | 175,356 | 145,827 | 72,963 | 15,242 | 18,391 | 106,596 | 27,703 |
| 1,657 | 1,289 | St. Marylebone and West Central District ... ... ... | 15,214 | 227,656 | 169,069 | 101,592 | 24,167 | 14,257 | 140,016 | 55,347 |
| 1,694 | 1,341 | Kensington and West District ... | 16,165 | 206,163 | 170,511 | 91,324 | 31,134 | 7,602 | 130,060 | 77,792 |
| 1,173 | 852 | Islington & North-West District | 10,367 | 178,317 | 104,432 | 67,615 | 11,283 | 7,487 | 86,385 | 35,624 |
| 1,541 | 1,258 | Stratford and East-End District | 21,330 | 333,387 | 251,750 | 97,621 | 36,044 | 4,047 | 137,712 | 32,846 |
| 9,844 | 7,605 | | 104,995 | 1,671,000 | 1,161,133 | 567,582 | 224,358 | 75,629 | 867,569 | 290,554 |

## Figures and Tables

**Figure f1:**
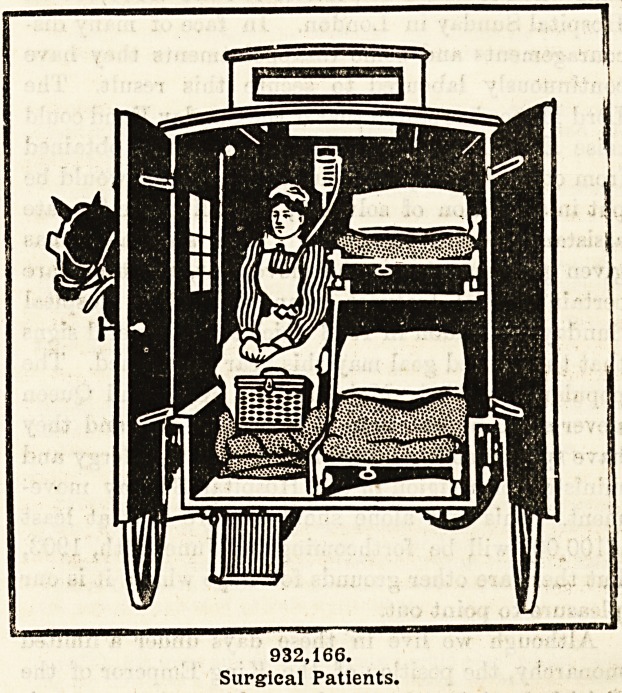


**Figure f2:**
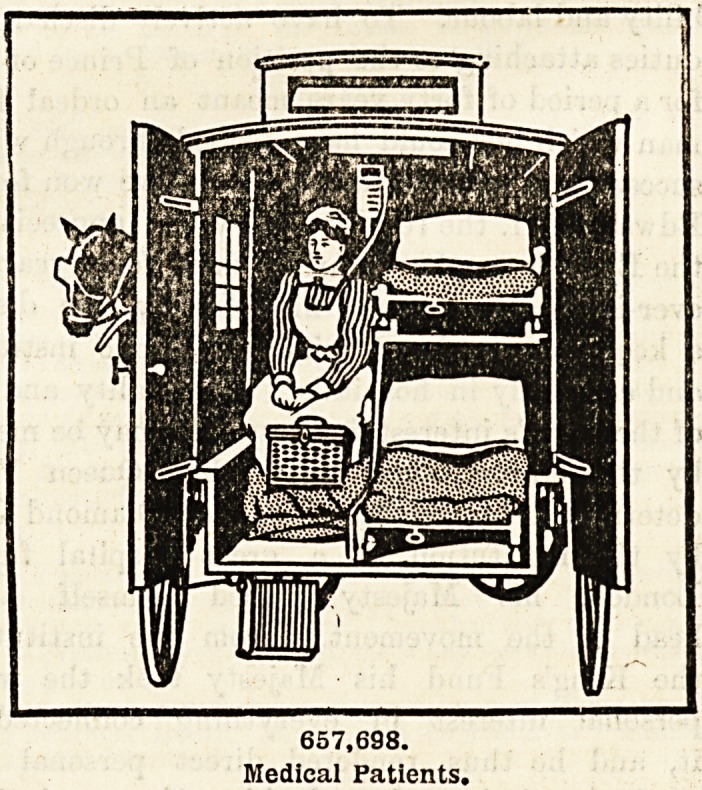


**Figure f3:**
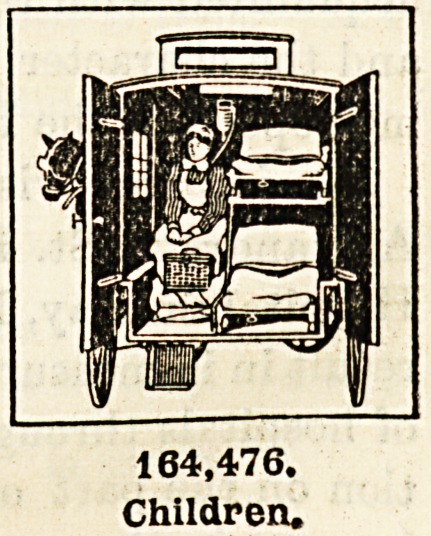


**Figure f4:**
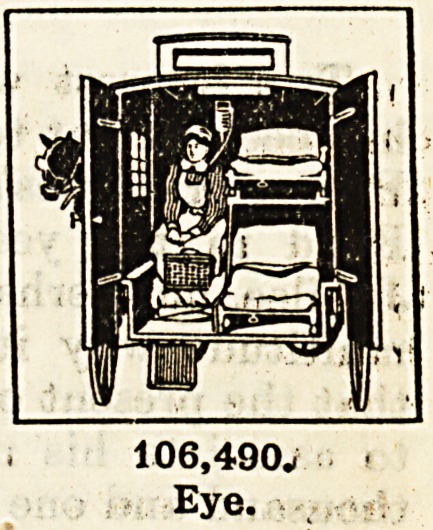


**Figure f5:**
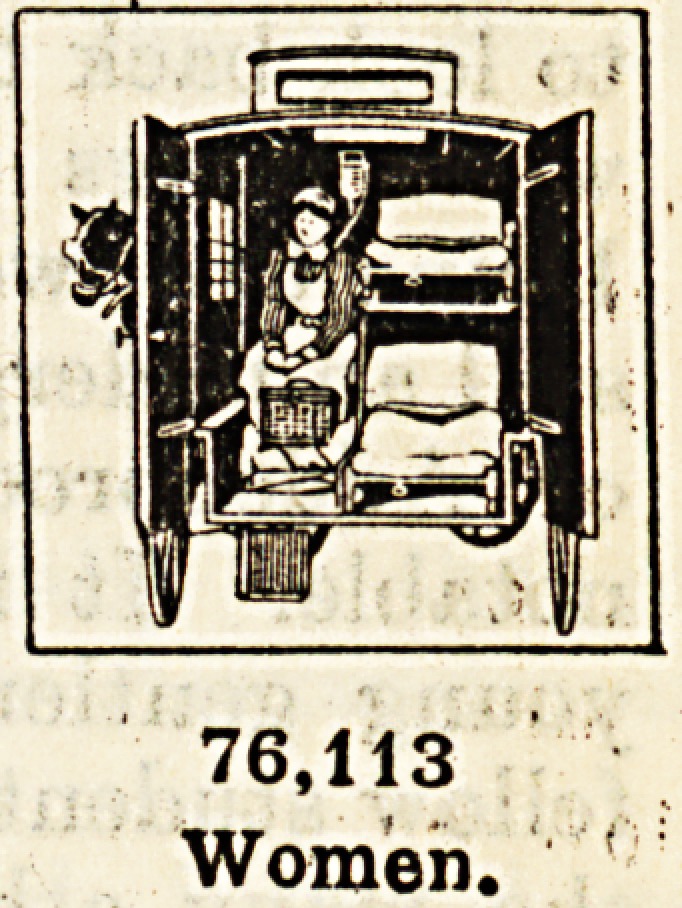


**Figure f6:**
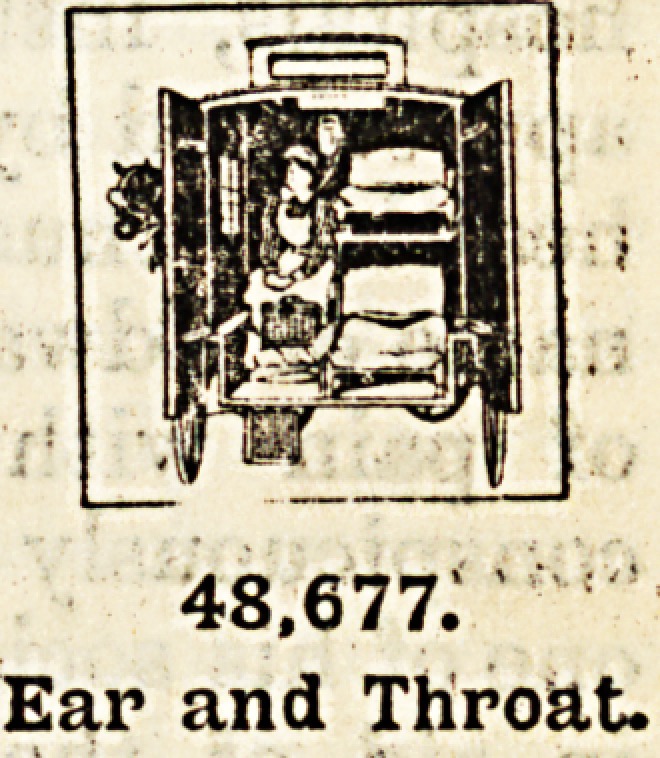


**Figure f7:**
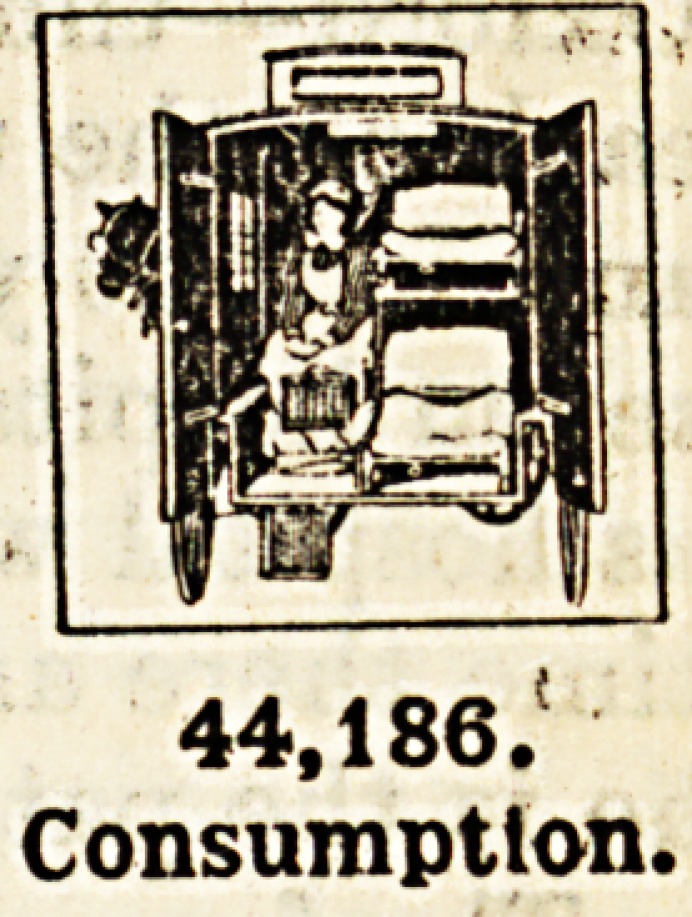


**Figure f8:**
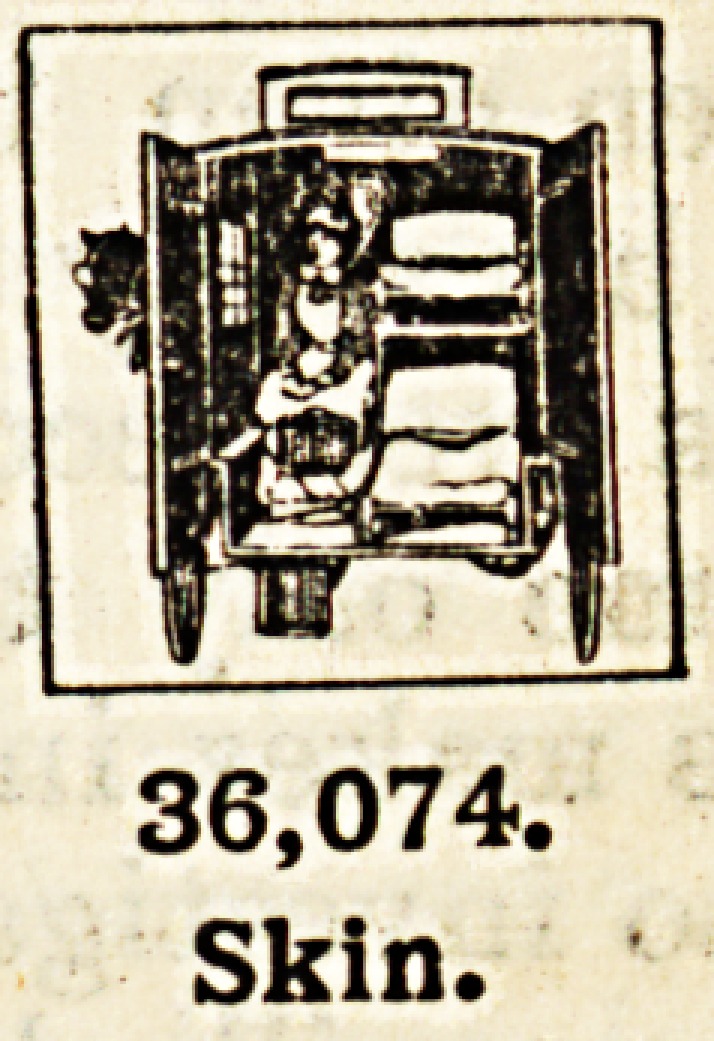


**Figure f9:**
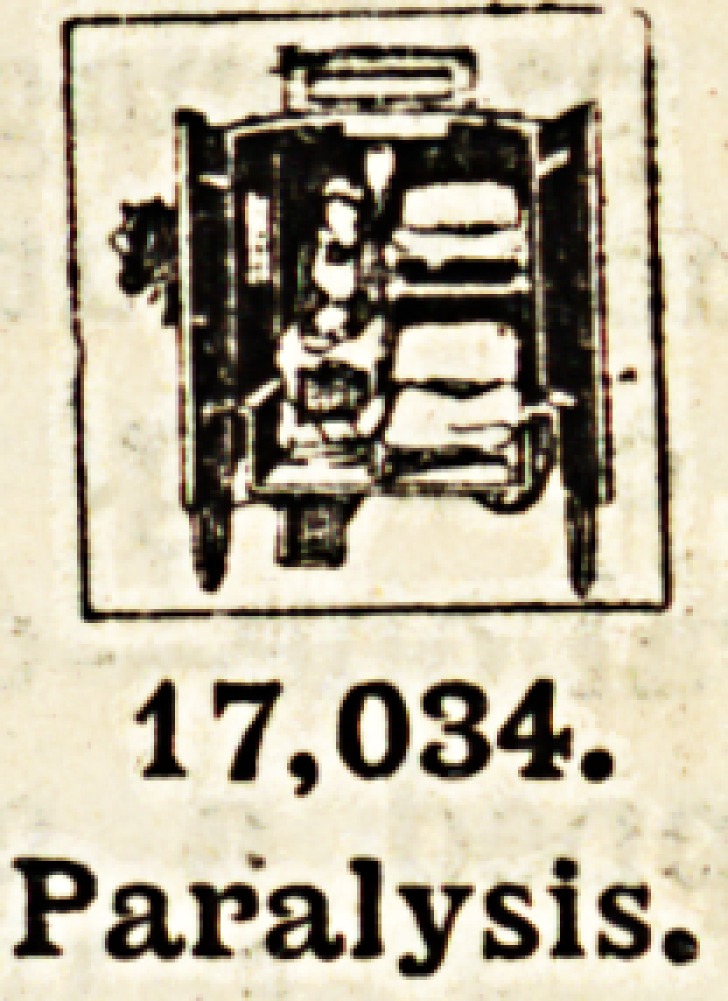


**Figure f10:**
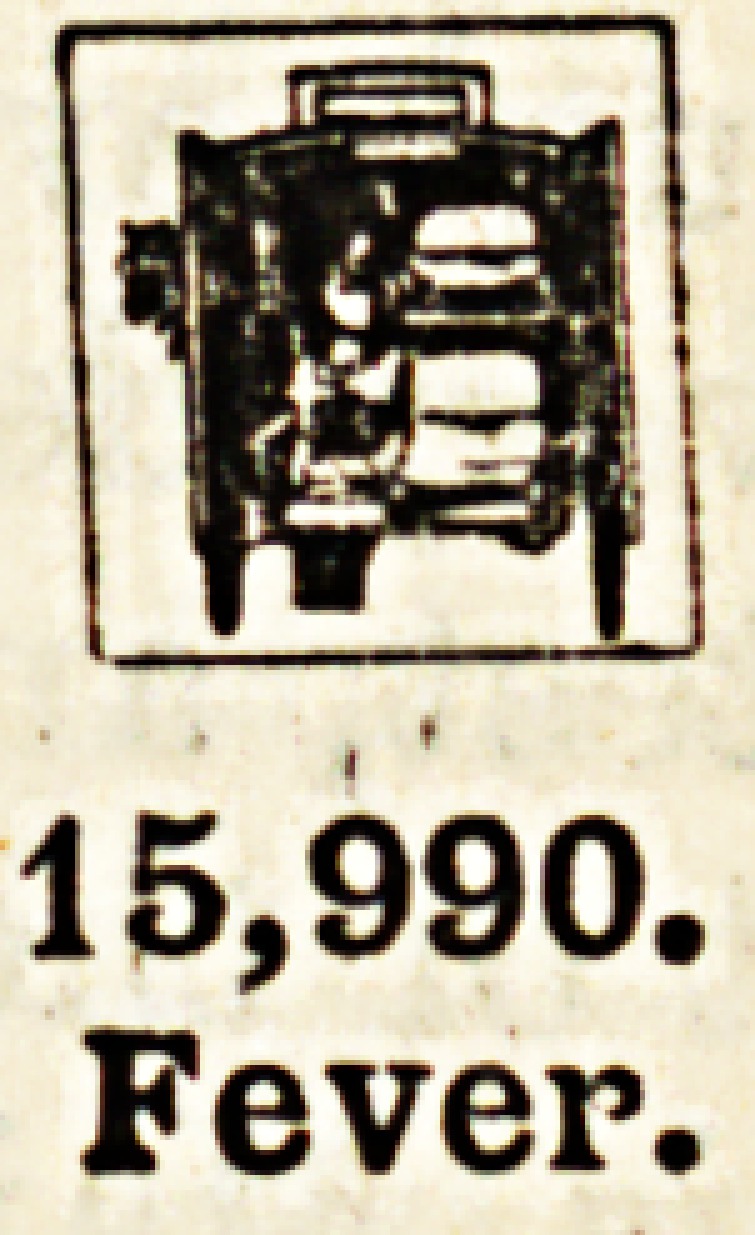


**Figure f11:**
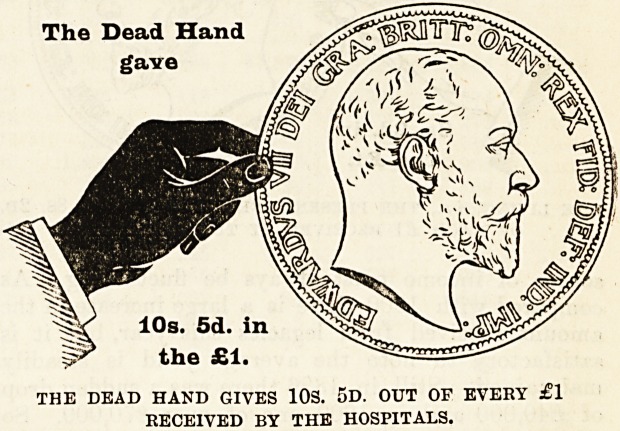


**Figure f12:**
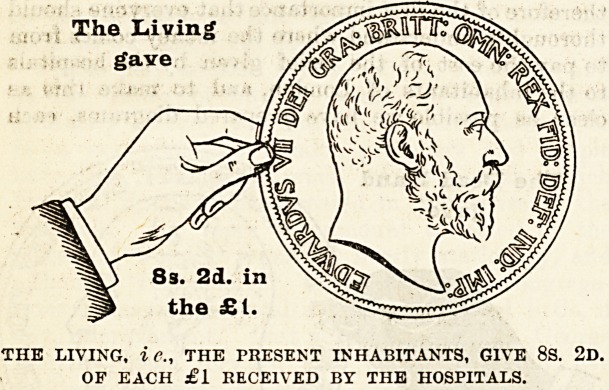


**Figure f13:**